# Endocan and Lumican in Relation to Cardiometabolic Risk in a Pediatric Overweight and Obese Cohort: A Cross-Sectional Study

**DOI:** 10.1155/2020/2102401

**Published:** 2020-08-17

**Authors:** Anca Bălănescu, Ioana Florentina Codreanu, Valentina Daniela Comanici, Iustina Violeta Stan, Eugenia Bălănescu, Paul Bălănescu

**Affiliations:** ^1^University of Medicine and Pharmacy “Carol Davila”, Bucharest - Pediatric Chair; 37 Dionisie Lupu Street, Bucharest, Romania; ^2^National Institute for Mother and Child Health “Alessandrescu-Rusescu”, 120 Lacul Tei Avenue, Bucharest, Romania; ^3^CDPC Clinical Immunology Department, Colentina Clinical Hospital, 19-21 Stefan cel Mare Street, Bucharest, Romania; ^4^Clinical Research Unit RECIF (Reseau d'Epidemiologie Clinique International Francophone), 19-21 Stefan cel Mare Street, Bucharest, Romania; ^5^University of Medicine and Pharmacy “Carol Davila”, Bucharest - Internal Medicine Chair; 37 Dionisie Lupu Street, Bucharest, Romania

## Abstract

The aim of the study was to evaluate serum Endocan and Lumican levels as biomarkers for pediatric Nonalcoholic Fatty Liver Disease (NAFLD) and to explore their associations with pediatric cardiometabolic risk factors. We conducted a cross-sectional study on 68 pediatric obese and overweight (O&O) patients. Ten healthy controls were recruited. Serum Lumican and Endocan levels were analyzed using ELISA kits. O&O patients had lower levels of Endocan compared to healthy controls (*p* < 0.001). There were no differences between serum Endocan levels in O&O patients with NAFLD and those without (*p* = 0.53). Patients considered having Nonalcoholic Steatohepatitis (NASH) had lower Endocan levels compared to O&O patients without NASH (*p* = 0.026). Patients with metabolic syndrome had lower levels of Endocan (*p* = 0.003). There were no significant differences between serum Lumican levels in O&O children compared to healthy controls. Lumican levels were higher in patients with hypertension (*p* = 0.04). In O&O patients, Lumican levels were negatively correlated with Endocan levels (*r* = −0.37, *p* = 0.002). Endocan seems a promising biomarker for the evaluation of pediatric NASH. Lumican was not confirmed as a biomarker for NAFLD in our cohort but was associated with higher arterial pressure. Low Endocan levels are accompanied by high serum Lumican levels, and this could be an early signature of cardiometabolic risk.

## 1. Introduction

Obesity and its alarming chronic complications are some of the major issues of nowadays health systems. Pediatric obesity is even more problematic as its prevalence is still increasing in many parts of the world [1] and alters adult life expectancy. High BMI as an obesity marker is long known to be strongly associated with atherosclerotic lesions even in the young [2]. As the cornerstone of obesity-related comorbidities is insulin resistance, those patients associate metabolic syndrome even from early ages. The hepatic manifestation of metabolic syndrome is known as Nonalcoholic Fatty Liver Disease (NAFLD). Nevertheless, this spectrum of liver disease troubles pediatricians worldwide as its rising prevalence surpasses even obesity's prevalence uprise in the last decade [3]. Furthermore, NAFLD involves two practical problems. Firstly, there is the problem of the invasive gold standard diagnosis method (liver biopsy) and secondly, lack of treatment. Additionally, NAFLD itself is related to cardiovascular risk, irrespective of the classical cardiometabolic risk factors [4].

Biomarkers are untiringly searched to bridge the gap in NAFLD accurate noninvasive diagnosis. From a simple mechanicist point of view, NAFLD is linked to insulin resistance, inflammation, and oxidative stress that ultimately cluster to determine liver fibrosis. So, molecules involved in those pathogenic chains could be suitable candidate biomarkers. There are some rigorous systematic reviews based on non-hypothesis-driven research [5] that claim particular circulating proteins' potential relevance in NAFLD screening and/or diagnosis. Two of those molecules are Endocan (Endothelial cell-Specific Molecule 1, ESM-1) and Lumican, but only few clinical studies address their applicability in NAFLD. The former is a proteoglycan well studied as an endothelial dysfunction biomarker in miscellaneous conditions like cancer, sepsis, pneumonia, pulmonary thromboembolism, chronic kidney disease, diabetes mellitus, preterm delivery-associated complications in neonates, and systemic sclerosis [6–9]. The latter is a small leucine-rich proteoglycan expressed in the extracellular matrix which regulates the process of collagen fibrillogenesis [10]. Also, recent results of fundamental studies establish the diet-dependent function of Lumican in obesity. When exposed to a high-fat diet, murine models showed that for adipose tissue expansion, an entire range of processes are triggered such as inflammation, angiogenesis, and extracellular matrix remodeling and Lumican and Endocan seem to play a key role [11].

The aim of the present study was to evaluate serum Endocan and Lumican levels as biomarkers for pediatric NAFLD and to explore their associations with pediatric cardiometabolic risk factors.

## 2. Materials and Methods

We conducted a cross-sectional study on 68 consecutive pediatric patients admitted in a single center (Pediatric Clinic of the National Institute for Mother and Child Health “Alessandrescu-Rusescu,” Bucharest) in a one-year period (January to December 2017). Those patients were part of the MR-PONy cohort (Metabolic and cardiovascular Risk factors in a Pediatric Overweight and obese population with or without NAFLD) whose characteristics were previously fully described in our previous studies [12, 13]. All eligible patients had not been treated prior to enrollment. Enrolled patients were overweight or obese children of 3 to 18 years old whose legal tutor agreed to take part in this observational study by signing the informed consent. Overweight was defined according to the CDC definition [14] as a value of body mass index (BMI) greater than the 85^th^ percentile for age and gender but below the 95^th^ percentile. Obesity was considered when the BMI value was above the 95^th^ percentile for age and gender.

We excluded all patients who had either genetic traits, endocrine disease, celiac and Wilson disease, or alpha-1-antitrypsin deficiency. The study was agreed by the local ethics committee and was conducted according to the Declaration of Helsinki upon ethical principles for medical research involving human subjects.

Ten lean gender- and age-matched healthy controls were recruited from patients that addressed to the Pediatric Clinic of the National Institute for Mother and Child Health “Alessandrescu-Rusescu,” Bucharest, but in the end proved not to have any infectious nor chronic disease.

In order to estimate sample size, we used data from previously published studies performed on serum Endocan levels in hepatic diseases. Based on anticipated 40% difference of Endocan levels at a 3 : 1 enrollment rate (overweight and obese children versus healthy controls), we anticipated that a minimum number of 40 patients (10 from the control group and 30 from the overweight and obese group) should be enrolled for 80% statistical power and 0.05 *α* error [15].

All patients included underwent clinical examination and complete history check at admission. Data about type 2 diabetes mellitus or obesity in first degree relatives were also recorded. Standardized anthropometric measurements (weight, height, BMI, waist to height ratio (WtHR), waist circumference, and midarm circumference) were assessed and recorded as a rough value and as a percentile value. All measurements were done according to “WHO STEPS Surveillance Manual: The WHO STEPwise Approach to Chronic Disease Risk Factor Surveillance/Noncommunicable Diseases and Mental Health, World Health Organization*”* [16].

Blood was drawn in order to evaluate lipid profile, glycemic status, liver function, and inflammatory status. A couple of atherogenic indexes were calculated: non-HDL cholesterol level and triglyceride to HDL cholesterol ratio. Also, the HOMA-IR (homeostatic model assessment insulin resistance) values were recorded. According to Calcaterra et al. [17], we used a cut-off value of 2.5 in prepubertal patients and of 4 in pubertal patients to define insulin resistance.

The presence of metabolic syndrome was evaluated. We defined metabolic syndrome according to IDF criteria [18], but we recorded criteria fulfillment also for the under 10-year-old patients.

All patients enrolled were evaluated for hepatic steatosis using 2D ultrasonography (Toshiba Aplio 300). NAFLD was considered when increased liver echogenicity in relation to the right kidney was noticed or when the ALAT levels were at least twofold greater than normal using the cut-off values proposed by Schwimmer et al. [19]. NASH (Nonalcoholic Steatohepatitis) was considered when ALAT levels were above 80 U/mL [20].

Blood was kept in -80 Celsius degrees freezer for further evaluation. Two proteoglycans of interest—Endocan and Lumican—were analyzed by the ELISA “sandwich” technique using commercial kits (Lunginnov, France, for Endocan and RayBiotech, USA, for Lumican). The limit of quantification for Endocan was 0.3 ng/mL with an intra-assay variation coefficient of 4.8% and an interassay variation coefficient of 7.59%. The sensitivity for Lumican assay was 0.1 ng/mL with an intra-assay variation coefficient < 10% and an interassay variation coefficient < 12%. Samples were analyzed in duplicate, and mean optical densities were used in order to determine the serum concentration of biomarkers of interest. Optical densities were read with an ELISA plate reader (Bio-Rad).

Data were analyzed with SPSS for Windows, version 16. Data were presented as mean and standard deviation if the distribution was normal and with median and interquartile range values if the distribution was nonnormal. Categorical variables were presented as percentage. For analyzing differences between groups, nonparametric tests were used for continuous variables. In order to evaluate the associations between outcomes (NASH, metabolic syndrome) and Endocan and Lumican, binary logistic regressions were performed after adjusting for variables that were also statistically significantly associated with the outcome of interest.

## 3. Results and Discussion

A number of 68 overweight and obese patients were included in the study. 35 (51.5%) of them were boys. The median age was 10 years (interquartile range 6). Metabolic syndrome prevalence according to IDF criteria was 36.8% (25 patients). NAFLD prevalence in the study cohort was 38.2% (26 patients), and 7 patients had NASH criteria (10.2%). Median HOMA-IR was 2.20 (2.87). Anthropometric assessment results and patients' characteristics are described in Tables 1 and 2, respectively. Study population characteristics were also fully described in a previously published study based on the MR-PONy cohort [12, 13].

Ten gender- and age-matched healthy controls were recruited (seven boys and three girls) with a median age of nine years (interquartile range 5). There was no difference in either gender distribution between controls and overweight and obese patients (*p* = 0.22, Fisher exact test) nor age (*p* = 0.77, Mann-Whitney *U* test).

**Figure 1:**
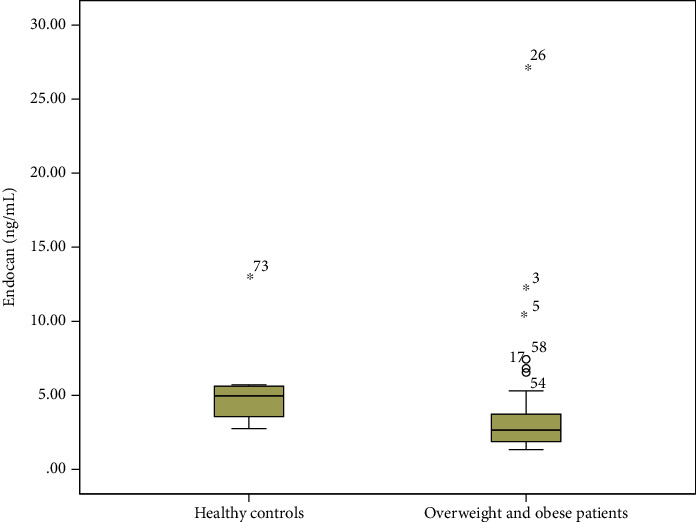
Serum Endocan levels were statistically significantly lower in overweight and obese patients compared to healthy controls (*p* < 0.001, Mann-Whitney *U* test).

**Figure 2:**
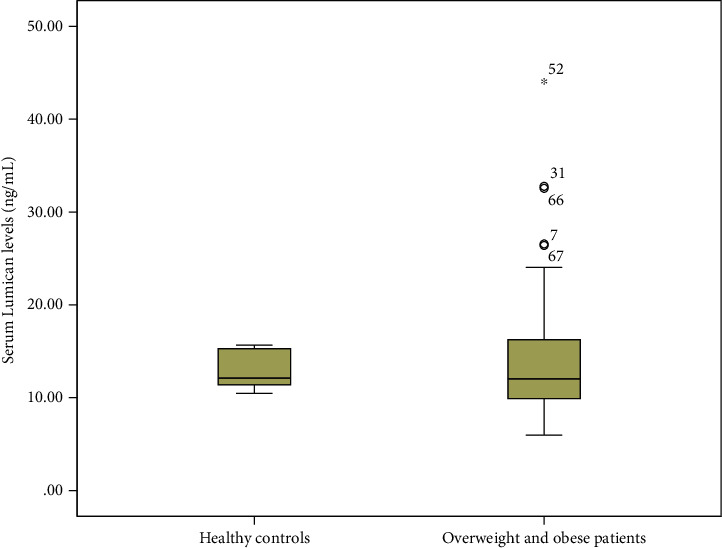
There were no differences between serum Lumican levels in overweight and obese patients and healthy controls (*p* = 0.60, Mann-Whitney *U* test).

Overweight and obese patients had statistically significant lower levels of Endocan compared to healthy controls (median 2.65 ng/mL (1.88) versus median 4.95 ng/mL (2.26), *p* < 0.001, Mann-Whitney *U* test, Figure 1). On the other hand, there were no statistically significant differences between serum Lumican levels in overweight and obese children compared to healthy controls (median 11.75 ng/mL (6.65) versus median 12.11 ng/mL (4.08), *p* = 0.60, Mann-Whitney *U* test, Figure 2).

**Figure 3:**
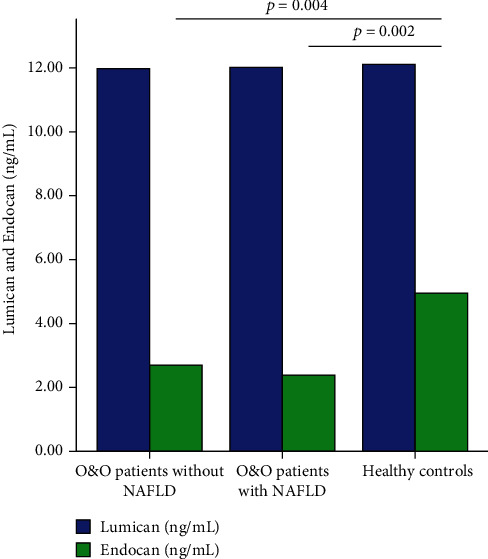
Serum Endocan and Lumican levels in overweight and obese patients without NAFLD, with NAFLD, and healthy controls. No differences were observed between Lumican levels, and only significant differences between groups were highlighted after performing post hoc Bonferroni correction.

There was found a difference between overweight and obese patients with NAFLD, overweight and obese patients without NAFLD, and healthy controls in serum Endocan levels (Kruskal-Wallis test, Figure 3, *p* = 0.006). Lower Endocan levels were observed in overweight and obese patients and NAFLD and non-NAFLD compared to healthy controls (*p* = 0.002, respectively, *p* = 0.004, Mann-Whitney *U* tests after Bonferroni post hoc corrections, Figure 3). No differences were observed for Lumican levels (Kruskal-Wallis test, *p* = 0.83).

Table 3 summarizes the differences between overweight and obese patients with NAFLD and overweight and obese patients without NAFLD.

There were no differences between serum Endocan levels in overweight and obese patients with NAFLD and those without (*p* = 0.53, Mann-Whitney *U*). Overweight and obese patients with NAFLD had higher age, higher waist percentile, higher BMI, triglycerides, triglyceride/HDL cholesterol ratio, ASAT, ALAT, insulin, and HOMA-IR values (Table 3).

Table 4 summarizes differences between overweight and obese patients with NASH and overweight and obese patients without NASH. Overweight and obese patients had higher ASAT, ALAT, and triglyceride/HDL cholesterol ratio compared to overweight and obese patients without NASH. Interestingly, the prevalence of obesity in NASH patients was lower compared to non-NASH patients.

**Figure 4:**
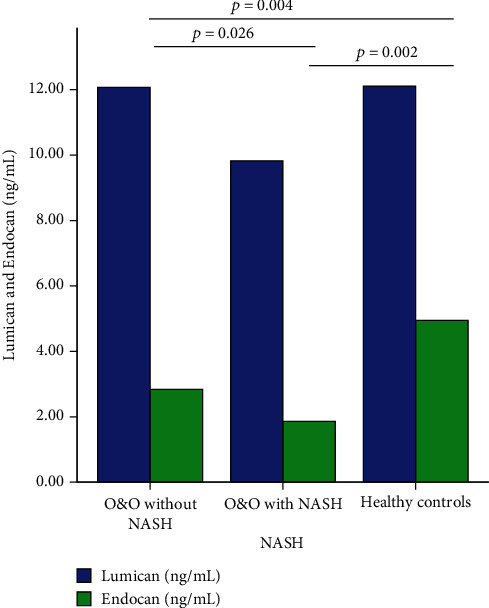
Serum Endocan and Lumican levels in overweight and obese patients without NASH, with NASH, and healthy controls. No differences were observed between Lumican levels, and only significant differences between groups were highlighted after performing post hoc Bonferroni correction.

There was found a difference between overweight and obese patients with NASH, overweight and obese patients without NASH, and healthy controls in serum Endocan levels (Kruskal-Wallis test, Figure 4, *p* = 0.001). Lower Endocan levels were observed in overweight and obese patients and NASH and non-NASH compared to healthy controls (*p* = 0.002, respectively, *p* = 0.004, Mann-Whitney *U* tests after Bonferroni post hoc corrections, Figure 4). No differences were observed for Lumican levels (Kruskal-Wallis test, *p* = 0.66).

**Figure 5:**
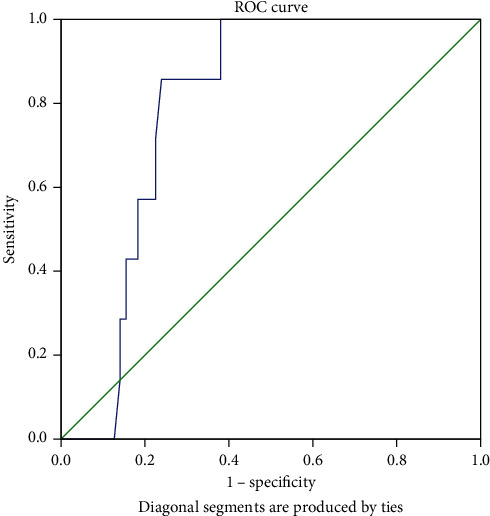
ROC curve of Endocan for NASH diagnosis (AUC = 0.76 (95% confidence interval 0.64-0.87)).

Patients considered having NASH had lower Endocan levels compared to overweight and obese patients without NASH (1.86 ng/mL (0.24) versus 2.83 ng/mL (1.82), *p* = 0.026, Mann-Whitney *U* test). When used as a diagnostic test for NASH, Endocan had AUC of 0.76 (95% confidence interval 0.64-0.87, Figure 5). At a cut-off value of 2.03 ng/mL, Endocan had 85.7% sensitivity and 72.1% specificity for NASH diagnosis. Nevertheless, in multivariate analysis (binary logistic regression), Endocan did not independently associate with NASH (OR = 0.38, 95% confidence interval 0.09-1.58) after adjustment for BMI percentile and triglyceride/HDL ratio.

Table 5 summarizes differences between overweight and obese patients with metabolic syndrome and overweight and obese patients without metabolic syndrome. The former had a higher prevalence of hypertension and female gender and also higher percentiles of systolic and diastolic pressure compared to the latter (Table 5).

**Figure 6:**
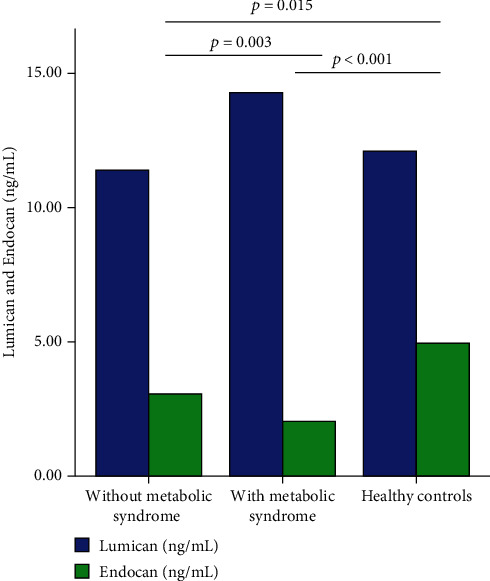
Serum Endocan and Lumican levels in overweight and obese patients without metabolic syndrome, with metabolic syndrome, and healthy controls. No differences were observed between Lumican levels, and only significant differences between groups were highlighted after performing post hoc Bonferroni correction.

There was found a difference between overweight and obese patients with metabolic syndrome, overweight and obese patients without metabolic syndrome, and healthy controls in serum Endocan levels (Kruskal-Wallis test, Figure 6, *p* < 0.001). Lower Endocan levels were observed in overweight and obese patients and metabolic syndrome and nonmetabolic syndrome compared to healthy controls (*p* = 0.015, respectively, *p* < 0.001, Mann-Whitney *U* tests after Bonferroni post hoc corrections, Figure 6). No differences were observed for Lumican levels (Kruskal-Wallis test, *p* = 0.11).

Overweight and obese patients that met IDF criteria for metabolic syndrome had statistically significant lower levels of Endocan (2.04 ng/mL (1.17) versus 3.06 ng/mL (2.03), *p* = 0.003, Mann-Whitney *U* test). Among all IDF criteria beside *sine qua non* criterion of central obesity (serum levels of triglycerides, serum HDL cholesterol levels, hypertension, and impaired glucose fasting), only patients that fulfilled triglyceride criteria had significantly lower Endocan levels (2.04 ng/mL (1.36) versus 2.92 ng/mL (1.96), *p* = 0.013, Mann-Whitney *U* test). Patients that fulfilled HDL cholesterol level criterion, impaired fasting glucose, and hypertension had a tendency for lower Endocan levels, although did not reach statistical significance (*p* = 0.112, *p* = 0.087, respectively, *p* = 0.097, Mann-Whitney *U* tests). Endocan levels did not associate with other clinical nor paraclinical characteristics of overweight and obese patients.

There was a tendency for patients that fulfilled IDF criteria for metabolic syndrome to have higher Lumican levels (14.29 ng/mL (7.68) versus 11.4 ng/mL (4.81), *p* = 0.06, Mann-Whitney *U* test). Among all IDF criteria, Lumican levels were statistically significantly higher in patients that fulfilled hypertension criterion (14.48 ng/mL (8.22) versus 11.45 ng/mL (4.59), *p* = 0.04, Mann-Whitney *U* test). There were no associations with triglyceride, HDL cholesterol, nor impaired fasting glucose criterion (*p* = 0.30, *p* = 0.36, respectively, *p* = 0.76, Mann-Whitney *U* tests). In multivariate analysis (binary logistic regression), Endocan and Lumican did not independently associate with metabolic syndrome after being adjusted for age, triglyceride/HDL cholesterol ratio, and systolic blood pressure percentile (OR = 0.93, 95% confidence interval 0.64-1.36 for Endocan and OR = 1.01, 95% confidence interval 0.89-1.13 for Lumican).

Serum Endocan levels negatively correlated with serum Lumican levels in overweight and obese patients (*r* = −0.37, *p* = 0.002, Spearman rho). However, this correlation is not observed in healthy controls (*r* = 0.17, *p* = 0.36). There is scarce data in the literature about Endocan and Lumican as biomarkers in NAFLD. Only few studies tackled Endocan as a biomarker in hepatic diseases with conflicting results. We found that Endocan levels were decreased in overweight and obese pediatric patients compared to healthy controls. The results are different from the only previously reported Endocan study in a pediatric population where there was no difference reported in Endocan levels between obese children and lean controls [21]. Nevertheless, in this previous study, there were mostly adolescents recruited (reported mean of age in obese subjects was approximatively 12 years with a small standard deviation of 0.4 years) while we included overweight and obese patients that were younger (median of 10 years). The authors also found a positive correlation of Endocan with age, younger patients having smaller circulating Endocan levels. There are previous reports in the literature that underline lower circulating levels of Endocan in obese adult population despite an increase in ESM-1 gene expression in adipocytes [22]. In this previous study, Endocan was negatively correlated with hsCRP (high-sensitivity C-reactive protein) and no correlation was found with HOMA-IR. The authors suggested that alongside with lower levels of circulating Endocan, cardiovascular risk could increase due to an increase in chronic inflammation (reflected by inverse correlation between Endocan and hsCRP) as Endocan seems to have a vasoprotective role. In our study, we did not find any correlation between Endocan and CRP levels in pediatric overweight and obese patients. Janke et al. suggested that insulin resistance could increase ESM-1 expression in adipocytes although circulating levels of Endocan were actually lower and no associations were found between circulating Endocan levels and ESM-1 gene expression in adipocytes. A recent study performed by Erman et al. [23] also found similar results in the adult population. In this study, the authors found lower Endocan levels in adult NAFLD patients and suggested that Endocan levels could be a predictive biomarker for metabolic syndrome. Our study comes as an external validation of these results on a pediatric population.

Endothelial dysfunction is associated with an increase in Endocan levels in the adult population [24]. A recent study performed on obese adolescents did not report a difference between biomarkers of endothelial dysfunction in patients with lower and higher BMI *Z*-scores [25]. As such, one could conclude that overweight and obese children might not yet develop biomarker changes of endothelial dysfunction. Therefore, Endocan levels would not increase in children due to endothelial dysfunction. Future studies that focus upon the pattern of Endocan secretion in children with and without endothelial dysfunction are warranted to sustain this hypothesis. Lower Endocan levels were found in overweight and obese patients that met IDF criteria for metabolic syndrome. More interestingly, patients with altered lipid levels had the lowest Endocan values, linking Endocan with altered lipid metabolism and cardiovascular risk in overweight and obese children.

Endocan levels in overweight and obese patients were lower in NASH patients compared to overweight and obese patients without NASH with a relatively good diagnostic value (AUC = 0.76 (95% confidence interval 0.64-0.87)) suggesting that it could be a biomarker for this condition. However, this result is different from previously reported literature, where Endocan levels were actually higher in NAFLD and the highest levels were observed in NASH patients compared to controls [26]. Nevertheless, this result was obtained from an adult not pediatric cohort and as Endocan was reported to be positively correlated with age, this could explain this conflicting result [21]. Also, there are reports that observed decreased Endocan levels in adult NAFLD [27]. Decreased Endocan levels were also associated with cardiac complications in adult cirrhotic patients [28]. All these previous data suggest that a decrease in Endocan levels could be linked with an increase in cardiac complications in chronic hepatic diseases. Future cohort studies that measure in a dynamic manner the pattern of Endocan are warranted in order to better answer conflicting results in the literature.

Lumican seemed a promising biomarker for NAFLD as it has been reported to be upregulated in previous proteomic studies from serum and biopsy samples in NAFLD patients [7]. However, to our knowledge, there are no studies in the literature that aimed to confirm circulating Lumican as a biomarker for NAFLD in overweight and obese pediatric patients. Serum Lumican levels were not different compared to healthy controls. Proteomic studies in adults identified Lumican as an important promoter of hepatic fibrosis [9]. Lumican overexpression in histological samples from adults was also associated with progressive NAFLD [29]. To our knowledge, there is no study on adult NAFLD population that evaluated serum Lumican levels, so we do not know if these intrahepatic modifications of Lumican are also reflected in circulation.

Overweight and obese patients that fulfilled hypertension IDF criterion for metabolic syndrome had higher Lumican levels. There are not many data about circulating Lumican in the literature, but one study observed that Lumican levels were higher in patients with acute aortic dissection compared to healthy controls due to exposure of interstitial collagenous matrices and Lumican release in the circulation [30]. A recent study reported higher serum Lumican levels in patients with carotid plaque compared to patients without carotid plaque. Interestingly, Lumican was the only small leucine-rich proteoglycan found to be upregulated in these patients even after adjusting for potential confounders. In this study, patients with higher systolic and diastolic arterial pressures had also higher Lumican levels [31].

All these findings underline the fact that Lumican could be a useful prognostic biomarker in pediatric high-risk patients for hypertensive cardiovascular-related complications of obesity. This biomarker is already expressed in overweight and obese pediatric patients that have metabolic syndrome and associate hypertension. This pattern seems to be also present in adulthood alongside with the development of atherosclerotic plaque due to hypertension. Circulating Lumican does not seem a useful biomarker for NAFLD itself but for atherosclerotic complications that are associated with metabolic syndrome. Future studies in the pediatric population that will evaluate serum Lumican levels alongside with carotid artery plaque will answer these questions.

An interesting finding in our study is the negative correlation between serum Endocan and Lumican levels in overweight and obese patients, a correlation that was not found in healthy controls. Endocan is known to have anti-inflammatory effects [32], and Lumican is associated with carotid atherosclerotic inflammation [31]. We could speculate that this negative correlation found in overweight and obese pediatric patients could reflect atherosclerotic plaque development in overweight and obese children and represent a subtle signature of cardiometabolic risk. Further studies focusing on this issue are warranted to verify if this pattern suggests higher cardiovascular risk in adulthood.

The present study comprises a series of limitations. Firstly, NAFLD was evaluated using ultrasonography and biochemical markers. Also, the study is cross-sectional and monocentric and does not comprise a large sample size. Also, it does not comprise a follow-up of patients, but the cross-sectional approach is the study design to be performed in order to conform candidate biomarkers. In addition, subclinical atherosclerosis was not evaluated.

## 4. Conclusions

In conclusion, Endocan seems a promising biomarker especially for evaluation of pediatric NASH. Lumican was not confirmed as a biomarker for NAFLD in overweight and obese patients but was associated with higher arterial pressure. Low Endocan levels are accompanied by high serum Lumican levels, and this could be an early signature of cardiometabolic risk in overweight and obese children.

## Figures and Tables

**Table 1:** Anthropometric assessment results in overweight and obese patients.

Variable	*N* = 68 patients
Boys (*n*, %)	*N* = 35 (51.5%)
Age (years)	10 (6)
Weight percentile	97^th^ (4)
Waist percentile	95^th^ (6)
Midarm circumference centile	95^th^ (8)
BMI (kg/m^2^)	24.5 (6.40)
BMI percentile	98^th^ (3)
BMI *Z*-score	2.03 (0.67)
WtHR	59.05 (7.53)

Data are presented as median (interquartile range). BMI: body mass index; WtHR: waist to height ratio.

**Table 2:** Overweight and obese patient biochemical characteristics.

Variable	*N* = 68 patients
Total cholesterol (mg/dL)	166 (49)
LDL cholesterol (mg/dL)	101 (38)
HDL cholesterol (mg/dL)	43.5 (19)
Triglyceride (mg/dL)	92 (70)
Non-HDL cholesterol (mg/dL)	122.5 (44)
Triglyceride/HDL cholesterol ratio	2.04 (2.23)
C-reactive protein (mg/dL)	0.34 (0.79)
Fasting glucose (mg/dL)	88.5 (10)
ASAT (UI/L)	23 (7)
ALAT (UI/L)	29 (14)
Alkaline phosphatase (UI/L)	239 (127)
Uric acid (mg/dL)	4.8 (2.4)
Obese (*n*, %)	59 (86.9%)
Insulin (mU/L)	10.3 (14.26)
HOMA-IR	2.20 (2.87)
Endocan (ng/mL)	2.65 (1.88)
Lumican (ng/mL)	11.75 (6.65)
Hypertension (*n*, %)	32 (47.1%)
Systolic blood pressure percentile	50^th^ (44)
Diastolic blood pressure percentile	50^th^ (45)

Data are presented as median (interquartile range). ALAT: alanine aminotransferase; ASAT: aspartate aminotransferase; HDL: high-density lipoprotein; LDL: low-density lipoprotein; HOMA-IR: Homeostatic Model Assessment of Insulin Resistance.

**Table 3:** Comparison between overweight and obese patients with NAFLD and overweight and obese patients without NAFLD.

Variable	NAFLD *N* = 26 patients	Non-NAFLD *N* = 48 patients	*p* value
Boys (*n*, %)	12 (46.2%)	23 (54.8%)	0.33
Age (years)	12 (7)	8.50 (5)	0.007
Weight percentile	97.5^th^ (4)	97^th^ (4)	0.22
Waist percentile	95^th^ (4)	95^th^ (7)	0.048
Midarm circumference centile	94^th^ (13)	95^th^ (7)	0.91
BMI (kg/m^2^)	29.5 (10.70)	24.05 (4.60)	0.004
BMI percentile	98^th^ (2)	98^th^ (2)	0.54
BMI *Z*-score	2.10 (0.58)	2.00 (0.75)	0.45
WtHR	61.61 (10.48)	58.6 (5.45)	0.06
Total cholesterol (mg/dL)	163.5 (36)	165 (57)	0.89
LDL cholesterol (mg/dL)	108 (27)	98 (48)	0.63
HDL cholesterol (mg/dL)	35 (18)	45 (17)	0.004
Triglyceride (mg/dL)	127.5 (100)	71 (44)	<0.001
Non-HDL cholesterol (mg/dL)	126.5 (32)	114.5 (48)	0.33
Triglyceride/HDL cholesterol ratio	3.51 (4.15)	1.76 (1.18)	<0.001
C-reactive protein (mg/dL)	0.26 (0.44)	0.46 (1.35)	0.13
Fasting glucose (mg/dL)	89.5 (13)	87 (9)	0.082
ASAT (UI/L)	26 (20)	22 (7)	0.027
ALAT (UI/L)	42.50 (39)	24.5 (11)	<0.001
Alkaline phosphatase (UI/L)	300 (77)	203 (129)	0.084
Uric acid (mg/dL)	5.05 (2.2)	4.40 (2.4)	0.43
Obese (*n*, %)	24 (92.3%)	35 (83.3%)	0.24
Insulin (mU/L)	16.89 (19.45)	7.71 (8.05)	0.015
HOMA-IR	3.69 (3.26)	1.67 (1.93)	0.006
Hypertension (*n*, %)	14 (53.8%)	18 (42.9%)	0.26
Systolic blood pressure centile	90^th^ (45)	50^th^ (41)	0.29
Diastolic blood pressure centile	70^th^ (45)	50^th^ (45)	0.48
Endocan (ng/mL)	2.38 (1.84)	2.69 (2.08)	0.53
Lumican (ng/mL)	12.02 (7.48)	11.97 (5.95)	0.76

Data are presented as median (interquartile range). BMI: body mass index; WtHR: waist to height ratio; ALAT: alanine aminotransferase; ASAT: aspartate aminotransferase; HDL: high-density lipoprotein; LDL: low-density lipoprotein; HOMA-IR: Homeostatic Model Assessment of Insulin Resistance. *p* values were computed with Mann-Whitney *U* tests for continuous variables and Fisher exact test for nominal variables.

**Table 4:** Comparison between overweight and obese patients with NASH and overweight and obese patients without NASH.

Variable	NASH patients *N* = 7	Non-NASH patients *N* = 61	*p* value
Boys (*n*, %)	4 (57.1%)	31 (50.8%)	1.00
Age (years)	10 (7)	10 (6)	0.28
Weight percentile	90^th^ (9)	97^th^ (4)	0.07
Waist percentile	95^th^ (6)	95^th^ (6)	0.82
Midarm circumference centile	75^th^ (49)	95^th^ (8)	0.07
BMI (kg/m^2^)	27.30 (10.70)	25 (5.95)	0.96
BMI percentile	96^th^ (10)	98^th^ (3)	0.062
BMI *Z*-score	1.80 (0.98)	2.04 (0.68)	0.088
WtHR	60.99 (12.56)	59.10 (6.80)	0.84
Total cholesterol (mg/dL)	169 (38)	162 (54)	0.67
LDL cholesterol (mg/dL)	108 (35)	99.5 (38)	0.52
HDL cholesterol (mg/dL)	34 (23)	43 (19)	0.08
Triglyceride (mg/dL)	134 (58)	91 (70)	0.07
Non-HDL cholesterol (mg/dL)	135 (33)	119 (44)	0.21
Triglyceride/HDL cholesterol ratio	3.53 (0.97)	2 (1.99)	0.034
C-reactive protein (mg/dL)	0.71 (0.91)	0.38 (0.92)	0.88
Fasting glucose (mg/dL)	87 (19)	89 (10)	0.70
ASAT (UI/L)	52 (33)	22 (7)	0.001
ALAT (UI/L)	92 (60)	28 (12)	<0.001
Alkaline phosphatase (UI/L)	232.5 (165)	243 (129)	0.81
Uric acid (mg/dL)	4.05 (3.9)	4.80 (2.3)	0.42
Obese (*n*, %)	4 (57.1%)	55 (90.2%)	0.045
Insulin (mU/L)	14.81 (21.38)	9.67 (13.74)	0.88
HOMA-IR	3.15 (4.53)	2.13 (2.86)	0.84
Endocan (ng/mL)	1.86 (0.24)	2.83 (1.82)	0.026
Lumican (ng/mL)	9.82 (11.35)	12.07 (6.24)	0.46
Hypertension (*n*, %)	3 (42.9%)	30 (49.2%)	1.00
Systolic blood pressure centile	50^th^ (45)	50^th^ (43)	0.89
Diastolic blood pressure centile	90^th^ (45)	50^th^ (45)	0.83

Data are presented as median (interquartile range). BMI: body mass index; WtHR: waist to height ratio; ALAT: alanine aminotransferase; ASAT: aspartate aminotransferase; HDL: high-density lipoprotein; LDL: low-density lipoprotein; HOMA-IR: Homeostatic Model Assessment of Insulin Resistance. *p* values were computed with Mann-Whitney *U* tests for continuous variables and Fisher exact test for nominal variables.

**Table 5:** Comparison between overweight and obese patients with metabolic syndrome and overweight and obese patients without metabolic syndrome according to IDF criteria.

Variable	Metabolic syndrome patients *N* = 25 patients	Without metabolic syndrome patients *N* = 43 patients	*p* value
Boys (%)	6 (24%)	29 (67.4%)	0.001
Age (years)	11 (6)	8 (6)	0.005
Weight percentile	97^th^ (5)	97^th^ (4)	0.47
Waist percentile	95^th^ (4)	95^th^ (4)	0.074
Midarm circumference centile	95^th^ (9)	95^th^ (7)	0.89
BMI (kg/m^2^)	28 (10.10)	23.97 (5.10)	0.005
BMI percentile	98^th^ (6)	98^th^ (11)	0.80
BMI *Z*-score	2.08 (0.79)	2 (0.71)	0.79
WtHR	61.17 (10.85)	59 (5.23)	0.30
Total cholesterol (mg/dL)	160 (38)	167 (53)	0.93
LDL cholesterol (mg/dL)	99 (33)	107.5 (46)	0.73
HDL cholesterol (mg/dL)	35 (8)	48 (15)	<0.001
Triglyceride (mg/dL)	157 (91)	70 (47)	<0.001
Non-HDL cholesterol (mg/dL)	128 (34)	114 (46)	0.13
Triglyceride/HDL cholesterol ratio	4.14 (3.31)	1.65 (1.11)	<0.001
C-reactive protein (mg/dL)	0.39 (0.54)	0.38 (1.40)	0.98
Fasting glucose (mg/dL)	89 (8)	88 (12)	0.71
ASAT (UI/L)	23 (9)	23 (7)	0.96
ALAT (UI/L)	31 (30)	26 (12)	0.07
Alkaline phosphatase (UI/L)	232.5 (146)	243 (111)	0.53
Uric acid (mg/dL)	5.20 (2.2)	4.30 (2.2)	0.16
Obese (*n*, %)	21 (84%)	38 (88.4%)	0.71
Insulin (mU/L)	14.81 (13.05)	7.75 (9.89)	0.083
HOMA-IR	3.15 (2.45)	1.69 (2.15)	0.085
Hypertension (%)	18 (72%)	14 (32.6%)	0.002
Systolic blood pressure percentile	90^th^ (49)	50^th^ (40)	0.026
Diastolic blood pressure percentile	90^th^ (45)	50^th^ (40)	0.040
Endocan (ng/mL)	2.04 (1.17)	3.06 (2.03)	0.003
Lumican (ng/mL)	14.29 (7.68)	11.40 (4.81)	0.06

Data are presented as median (interquartile range). BMI: body mass index; WtHR: waist to height ratio; ALAT: alanine aminotransferase; ASAT: aspartate aminotransferase; HDL: high-density lipoprotein; LDL: low-density lipoprotein; HOMA-IR: Homeostatic Model Assessment of Insulin Resistance. *p* values were computed with Mann-Whitney *U* tests for continuous variables and Fisher exact test for nominal variables.

## Data Availability

The data used to support the findings of this study are available from the corresponding author upon request.

## References

[1] Ogden C. L., Carroll M. D., Lawman H. G. (2016). Trends in obesity prevalence among children and adolescents in the United States, 1988–1994 through 2013–2014. *JAMA*.

[2] Berenson G. S., Srinivasan S. R., Bao W., Newman W. P., Tracy R. E., Wattigney W. A. (1998). Association between multiple cardiovascular risk factors and atherosclerosis in children and young Adults. *The New England Journal of Medicine*.

[3] Welsh J. A., Karpen S., Vos M. B. (2013). Increasing prevalence of nonalcoholic fatty liver disease among United States adolescents, 1988–1994 to 2007–2010. *The Journal of Pediatrics*.

[4] Tana C., Ballestri S., Ricci F. (2019). Cardiovascular risk in non-alcoholic fatty liver disease: mechanisms and therapeutic implications. *International Journal of Environmental Research and Public Health*.

[5] Lădaru A., Bălănescu P., Stan M., Codreanu I., Anca I. A. (2015). Candidate proteomic biomarkers for non-alcoholic fatty liver disease (steatosis and non-alcoholic steatohepatitis) discovered with mass-spectrometry: a systematic review. *Biomarkers*.

[6] Kali A., Shetty K. S. R. (2014). Endocan: a novel circulating proteoglycan. *Indian Journal of Pharmacology*.

[7] Bălănescu P., Lădaru A., Bălănescu E., Voiosu T., Baicus C., Dan G. A. (2016). Endocan, novel potential biomarker for systemic sclerosis: results of a pilot study. *Journal of Clinical Laboratory Analysis*.

[8] Kuluöztürk M., İn E., İlhan N. (2019). Endocan as a marker of disease severity in pulmonary thromboembolism. *The Clinical Respiratory Journal*.

[9] Balamir I., Ates I., Topcuoglu C., Turhan T. (2017). Association of endocan, ischemia-modified albumin, and hsCRP levels with endothelial dysfunction in type 2 diabetes mellitus. *Angiology*.

[10] Krishnan A., Li X., Kao W. W. Y. (2012). Lumican, an extracellular matrix proteoglycan, is a novel requisite for hepatic fibrosis. *Laboratory Investigation*.

[11] Wolff G., Taranko A. E., Meln I. (2019). Diet-dependent function of the extracellular matrix proteoglycan lumican in obesity and glucose homeostasis. *Molecular Metabolism*.

[12] Bălănescu A., Bălănescu P., Comănici V. (2018). Lipid profile pattern in pediatric overweight population with or without NAFLD in relation to IDF criteria for metabolic syndrome: a preliminary study. *Romanian Journal of Internal Medicine*.

[13] Bălănescu A., Stan I., Codreanu I., Comănici V., Bălănescu E., Bălănescu P. (2019). Circulating Hsp90 isoform levels in overweight and obese children and the relation to nonalcoholic fatty liver disease: results from a cross-sectional study. *Disease Markers*.

[14] March 2019, https://www.cdc.gov/obesity/childhood/defining.html

[15] https://clincalc.com/stats/samplesize.aspx

[16] March 2019, https://apps.who.int/iris/handle/10665/43376

[17] Calcaterra V., Klersy C., Muratori T. (2008). Prevalence of metabolic syndrome (MS) in children and adolescents with varying degrees of obesity. *Clinical Endocrinology*.

[18] Zimmet P., Alberti K. G. M. M., Kaufman F. (2007). The metabolic syndrome in children and adolescents – an IDF consensus report. *Pediatric Diabetes*.

[19] Schwimmer J. B., Dunn W., Norman G. J. (2010). SAFETY study: alanine aminotransferase cutoff values are set too high for reliable detection of pediatric chronic liver disease. *Gastroenterology*.

[20] Vajro P., Lenta S., Socha P. (2012). Diagnosis of nonalcoholic fatty liver disease in children and adolescents: position paper of the ESPGHAN Hepatology Committee. *Journal of Pediatric Gastroenterology and Nutrition*.

[21] Ustyol A., Aycan Ustyol E., Gurdol F., Kokali F., Bekpinar S. (2017). P-selectin, endocan, and some adhesion molecules in obese children and adolescents with non-alcoholic fatty liver disease. *Scandinavian Journal of Clinical and Laboratory Investigation*.

[22] Janke J., Engeli S., Gorzelniak K. (2006). Adipose tissue and circulating endothelial cell specific molecule-1 in human obesity. *Hormone and Metabolic Research*.

[23] Erman H., Beydogan E., Cetin S. I., Boyuk B. (2020). Endocan: a biomarker for hepatosteatosis in patients with metabolic syndrome. *Mediators of Inflammation*.

[24] Oktar S. F., Guney I., Eren S. A. (2018). Serum endocan levels, carotid intima-media thickness and microalbuminuria in patients with newly diagnosed hypertension. *Clinical and Experimental Hypertension*.

[25] Murni I. K., Sulistyoningrum D. C., Susilowati R., Julia M. (2020). Risk of metabolic syndrome and early vascular markers for atherosclerosis in obese Indonesian adolescents. *Paediatrics and International Child Health*.

[26] Dallio M., Masarone M., Caprio G. G. (2020). Endocan serum levels in patients with non-alcoholic fatty liver disease with or without type 2 diabetes mellitus: a pilot study. *Journal of Gastrointestinal and Liver Diseases*.

[27] Tok D., Ekiz F., Basar O., Coban S., Ozturk G. (2014). Serum endocan levels in patients with chronic liver disease. *International Journal of Clinical and Experimental Medicine*.

[28] Voiosu A. M., Bălănescu P., Daha I. (2018). The diagnostic and prognostic value of serum endocan in patients with cirrhotic cardiomyopathy. *Romanian Journal of Internal Medicine*.

[29] Charlton M., Viker K., Krishnan A. (2009). Differential expression of lumican and fatty acid binding protein-1: new insights into the histologic spectrum of nonalcoholic fatty liver disease. *Hepatology*.

[30] Gu G., Wan F., Xue Y. (2016). Lumican as a novel potential clinical indicator for acute aortic dissection: a comparative study, based on multi-slice computed tomography angiography. *Experimental and Therapeutic Medicine*.

[31] Yang Y., Wu Q. H., Li Y., Gao P. J. (2018). Association of SLRPs with carotid artery atherosclerosis in essential hypertensive patients. *Journal of Human Hypertension*.

[32] Gaudet A., Portier L., Prin M. (2019). Endocan regulates acute lung inflammation through control of leukocyte diapedesis. *Journal of Applied Physiology*.

